# Assessing the external validity of algorithms to estimate EQ-5D-3L from the WOMAC

**DOI:** 10.1186/s12955-016-0547-y

**Published:** 2016-10-04

**Authors:** Aliasghar A. Kiadaliri, Martin Englund

**Affiliations:** 1 Department of Clinical Sciences Lund, Orthopaedics, Clinical Epidemiology Unit, Lund University, Lund, Sweden; 2 Health Services Management Research Center, Institute for Futures Studies in Health, Kerman University of Medical Sciences, Kerman, Iran; 3 Clinical Epidemiology Research and Training Unit, Boston University School of Medicine, Boston, MA USA; 4 Clinical Epidemiology Unit, Skåne University Hospital, Klinikgatan 22, SE-221 85 Lund, Sweden

**Keywords:** Mapping algorithms, WOMAC, EQ-5D-3L, Knee pain, Knee osteoarthritis, External validity

## Abstract

**Background:**

The use of mapping algorithms have been suggested as a solution to predict health utilities when no preference-based measure is included in the study. However, validity and predictive performance of these algorithms are highly variable and hence assessing the accuracy and validity of algorithms before use them in a new setting is of importance. The aim of the current study was to assess the predictive accuracy of three mapping algorithms to estimate the EQ-5D-3L from the Western Ontario and McMaster Universities Osteoarthritis Index (WOMAC) among Swedish people with knee disorders. Two of these algorithms developed using ordinary least squares (OLS) models and one developed using mixture model.

**Methods:**

The data from 1078 subjects mean (SD) age 69.4 (7.2) years with frequent knee pain and/or knee osteoarthritis from the Malmö Osteoarthritis study in Sweden were used. The algorithms’ performance was assessed using mean error, mean absolute error, and root mean squared error. Two types of prediction were estimated for mixture model: weighted average (WA), and conditional on estimated component (CEC).

**Results:**

The overall mean was overpredicted by an OLS model and underpredicted by two other algorithms (*P* < 0.001). All predictions but the CEC predictions of mixture model had a narrower range than the observed scores (22 to 90 %). All algorithms suffered from overprediction for severe health states and underprediction for mild health states with lesser extent for mixture model. While the mixture model outperformed OLS models at the extremes of the EQ-5D-3D distribution, it underperformed around the center of the distribution.

**Conclusions:**

While algorithm based on mixture model reflected the distribution of EQ-5D-3L data more accurately compared with OLS models, all algorithms suffered from systematic bias. This calls for caution in applying these mapping algorithms in a new setting particularly in samples with milder knee problems than original sample. Assessing the impact of the choice of these algorithms on cost-effectiveness studies through sensitivity analysis is recommended.

**Electronic supplementary material:**

The online version of this article (doi:10.1186/s12955-016-0547-y) contains supplementary material, which is available to authorized users.

## Background

Quality-adjusted life years (QALYs) is a very common outcome measure applied in cost-utility analysis. QALY combines both health-related quality of life (HRQoL) and survival into a single metric, where survival is weighted by health utilities. These health utilities are cardinal values ranging from 1 (equivalent to full health) to zero (equivalent to death) with possible negative values for health states worse than death [1]. The health utilities can be elicited using direct method (e.g., standard gamble, time trade-off) or indirect method applying a generic preference-based measure of HRQoL. The EuroQol five-dimension (EQ-5D) is a widely used generic preference-based measure of HRQoL to elicit health utilities.

However, many clinical studies continue to use condition-specific measures of HRQoL that are non-preference-based and have limited use in estimating health utilities and in cost-utility analyses. In response to this, the use of mapping algorithms have been suggested as a solution to predict health utilities from these condition-specific non-preference-based measures when no preference-based measure is included in the study [2, 3]. However, validity and predictive performance of these algorithms are highly variable [2] and concerns on their reliability and accuracy had been raised [4]. It has been shown that different algorithms can result in different incremental cost-effectiveness ratios and possibly discrepant funding decisions [5]. These concerns imply that assessing the accuracy and validity of algorithms before use them in a new setting is of importance. This assessment is known as external validity and determine to what extent the results of a mapping algorithm can be generalized/applied to other people/setting. It should be noted that in assessing external validity of any prediction model, one should distinguish between model reproducibility and model transportability [6]. The former refers to model performance in a new sample with similar case mix as the original sample, while the latter refers to model performance in a new sample with different case mix compared with the original sample [6].

In the study of knee pain and knee osteoarthritis (OA), the Western Ontario and McMaster Universities Osteoarthritis Index (WOMAC) is a commonly used as disease-specific non-preference-based measure [7]. To enable using the WOMAC in cost-utility analysis, three algorithms have been developed to estimate EQ-5D-3L from the WOMAC [8–10] and had been used in cost-utility analyses [11, 12]. To our best knowledge, no previous study compared the predictive accuracy of these algorithms in an external sample of people with knee pain and OA. To fill this knowledge gap, the aim of the current study was to compare the predictive accuracy of these algorithms in a large sample of Swedish patients with knee pain and/or knee OA who answered to both EQ-5D-3L and WOMAC questionnaires. In the current study, we investigated whether EQ-5D-3L can be reliably predicted from WOMAC using current algorithms. This is an important question since presence of any difference between actual and predicted values can have crucial impact on cost-utility analyses and funding decisions.

## Methods

### Data

**Fig. 1:**
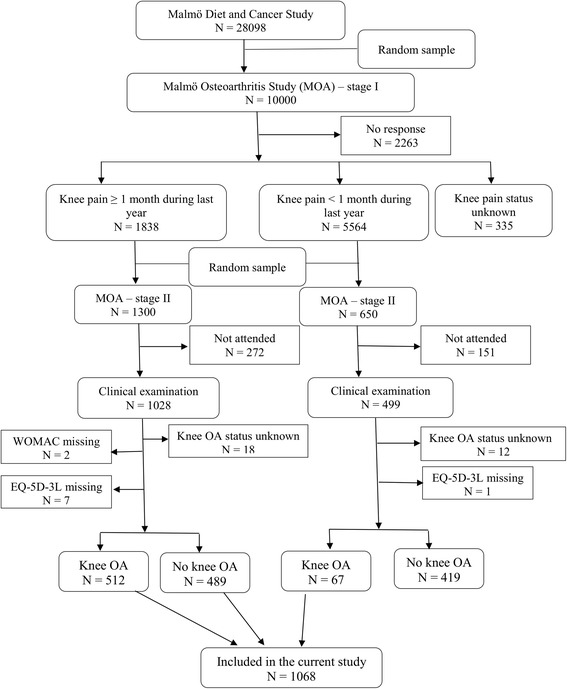
Flow diagram of the study design

We used the data from the Malmö OA (MOA) study originating from the Malmö Diet and Cancer Study. In the first stage of the MOA study, a postal questionnaire about knee pain was sent to a random sample of 10 000 subjects from the Malmö Diet and Cancer Study who were still alive and resident in the Malmö area in 2007. Respondents were asked about whether they have had knee pain during the previous 12 months and its duration (<1 week, 1–4 weeks, 1–3 months, >3 months). Subjects with pain in one or both knees in the past 12 months and duration of minimum 1 month were classified as having knee pain. In the second stage of the MOA, a random sample of 1 300 subjects with knee pain and 650 subjects without knee pain were invited to a clinical visit and radiographic examination [13]. A total of 1 527 subjects participated in the second stage and responded to the EuroQol-5D-3L (EQ-5D-3L) and the Knee injury and Osteoarthritis Outcome Score (KOOS) questionnaires. For this study we used the data on 494 subjects with knee pain without knee OA and 584 subjects with knee OA (either clinical [14] or/and radiographic [15, 16]). The subjects with neither knee pain nor knee OA (*n* = 419) were not included as the algorithms were not intended to be applied for these people. An addition of 40 subjects were excluded due to missing on knee OA status (*n* = 30), EQ-5D-3L (*n* = 8), and WOMAC (*n* = 2) questionnaires (Fig. 1).

### HRQoL measures

The EQ-5D-3L is a generic preferences-based health measure covering five attributes: mobility, self-care, usual activities, pain/discomfort, and anxiety/depression. Each attribute has three levels: no problems, some or moderate problems, and severe problems, resulting in 243 (3^5^) possible health states [17]. The responses to these attributes were weighted using the UK [18] time trade-off value set to calculate observed UK EQ-5D-3L index scores.

The WOMAC is a disease-specific questionnaire consisting of three domains: “pain”, “stiffness”, and “physical function” [7]. Since the KOOS was developed based on the WOMAC LK 3.0 questionnaire, the WOMAC subscale scores can be calculated from the KOOS. Using responses to questions P5-P9 (5 questions) from KOOS-pain subscale, S6-S7 (2 questions) from KOOS- other symptoms subscale, and A1-A17 (17 questions) KOOS-ADL subscale, we calculated scores for pain, stiffness and physical functions subscales of the WOMAC, respectively. The response options for these questions are none (0), mild (1), moderate (2), severe (3) or extreme (4). This means that scores range 0–20 for pain subscale, 0–8 for stiffness subscale, 0–68 for physical function subscale, and 0–96 for total WOMAC score (higher scores indicate more problems).

### Algorithms

Three algorithm are currently available to estimate the EQ-5D-3L index scores from the WOMAC. The algorithm developed by Barton et al. [8] used data on 348 individuals with knee pain in the UK. The mean UK EQ-5D-3L index score was 0.557 and the mean WOMAC subscales were as follow: 7.76 for pain, 3.91 for stiffness, and 27.89 for physical function. In this algorithm, the UK EQ-5D-3L index scores were modelled by ordinary least squares (OLS) regression (EQ-5D-3L index score = −0.3474012785 − 0.0005977709*WOMAC total score − 0.0001081560*WOMAC total score^2^ + 0.0326027536*age − 0.0002352456*age^2^ + 0.0475889687*sex) [8]. The algorithm developed by Xie et al. [9] included a sample of 258 subjects with knee OA in Singapore. In this study the Japanese value set [19] was used to calculate the EQ-5D-3L index scores. The mean Japanese EQ-5D-3L index score was 0.62 with following scores on the WOMAC subscales: 6.64 on pain, 3.12 on stiffness, and 26.24 on physical function. The final model was developed using the OLS model (EQ-5D-3L index score = 0.83414 − 0.00166*WOMAC pain score − 0.00092*WOMAC stiffness score − 0.00330*WOMAC function score). The algorithm developed by Wailoo et al. [10] used 7 072 observations from 1 768 subjects with knee or hip OA from three hospitals in Spain. These subjects had a mean UK EQ-5D-3L index score of 0.29 with scores of 11.37, 4.70, and 43.85 on the WOMAC pain, stiffness, and physical function, respectively. In this algorithm, a random effects adjusted limited dependent variable mixture model based on a distribution specific to the characteristics of EQ-5D was applied. This model was used to account for the characteristics of EQ-5D-3L data including the right and left bounding, a mass of observations at 1.0 (full health), a large gap between full health and the next feasible EQ-5D-3L value, and multimodality of the distribution [20]. The final algorithm had a mixture of five components and these components and probability of their membership were estimated using WOMAC pain, WOMAC stiffness, WOMAC function, their quadratic terms, age, and sex [10]. We used the EQ-5D calculator provided by Wailoo et al. [10] to estimate EQ-5D-3L index scores from the WOMAC (the calculator is available at: http://hqlo.biomedcentral.com/articles/10.1186/1477-7525-12-37).

### Statistical analysis

The algorithms were applied to the WOMAC responses and the EQ-5D-3L index scores were predicted (in follow, we identified each algorithm by the name of the first author). It should be noted that two types of predictions can be estimated from mixture models [21]: 1) “weighted average (WA)” as the sum of predictions for each component multiplied by probability of component membership, and 2) “conditional on estimated component (CEC)” which is equal to the prediction for the component with the maximum membership probability. We calculated both these values for the Wailoo algorithm [10].

The predictive accuracy was assessed by evaluating the scatter plots of the observed scores versus prediction error, and calculation of the mean error, the mean absolute error (MAE), the root mean squared error (RMSE), and the proportion of absolute errors greater than 5 %, 10 %, and 25 % of observed scores. The MAE is the mean of absolute differences between the observed and predicted EQ-5D-3L index scores, whilst the RMSE is defined as the squared root of the mean of squared differences between the observed and predicted EQ-5D-3L index scores. These are recommended and widely used measures in assessing the performance of mapping algorithms [2] and smaller values of MAE/RMSE show better model performance. In addition, the performance of the algorithms according to severity of health states (ranked by EQ-5D-3L index score) and knee problems (ranked by total WOMAC score) were assessed. All analyses were performed in Microsoft Excel and STATA 13 (StataCorp LP, College Station, TX, USA).

## Results

The mean (SD) age and body mass index of the subjects included in the study were 69.4 (7.2) years and 28.2 (5.0), respectively, and 66.3 % were women (Table 1). Of these subjects, 45.8 % had knee pain without knee OA, 6.3 % had knee OA without knee pain, and 47.9 % had knee OA with knee pain. The mean (SD) scores on WOMAC-pain, stiffness, and physical function were 5.92 (4.05), 2.63 (1.91), and 21.86 (14.70), respectively. The mean (SD) EQ-5D-3L index score was 0.718 (0.214). A total of 48 out of 243 possible EQ-5D-3L health states were observed in the study sample and four health states 11121, 21121, 11111, and 11122 constitute 68.2 % of the observed EQ-5D-3L health states (Additional file 1: Table S1).

**Table 1:** Characteristics of the study sample

	Subjects with frequent knee pain and no knee OA (*n* = 489)	Subject with knee OA (with or without frequent knee pain) (*n* = 579)
Women, %	70.8	62.5
Age, years (SD)	68.2 (7.2)	70.4 (7.0)
Body mass index (SD)	27.4 (4.4)	28.9 (5.4)
Smoking, %		
Never	42.2	42.4
Current	14.6	12.3
Ex-smoker	43.2	45.3
Comorbidity, %		
None	12.9	14.4
Single	30.9	25.0
Multiple	56.2	60.6
WOMAC scores, mean (SD)		
Pain	4.7 (3.6)	7.0 (4.1)
Stiffness	2.0 (1.7)	3.2 (1.9)
Physical function	17.3 (13.5)	25.8 (14.5)

**Fig. 2:**
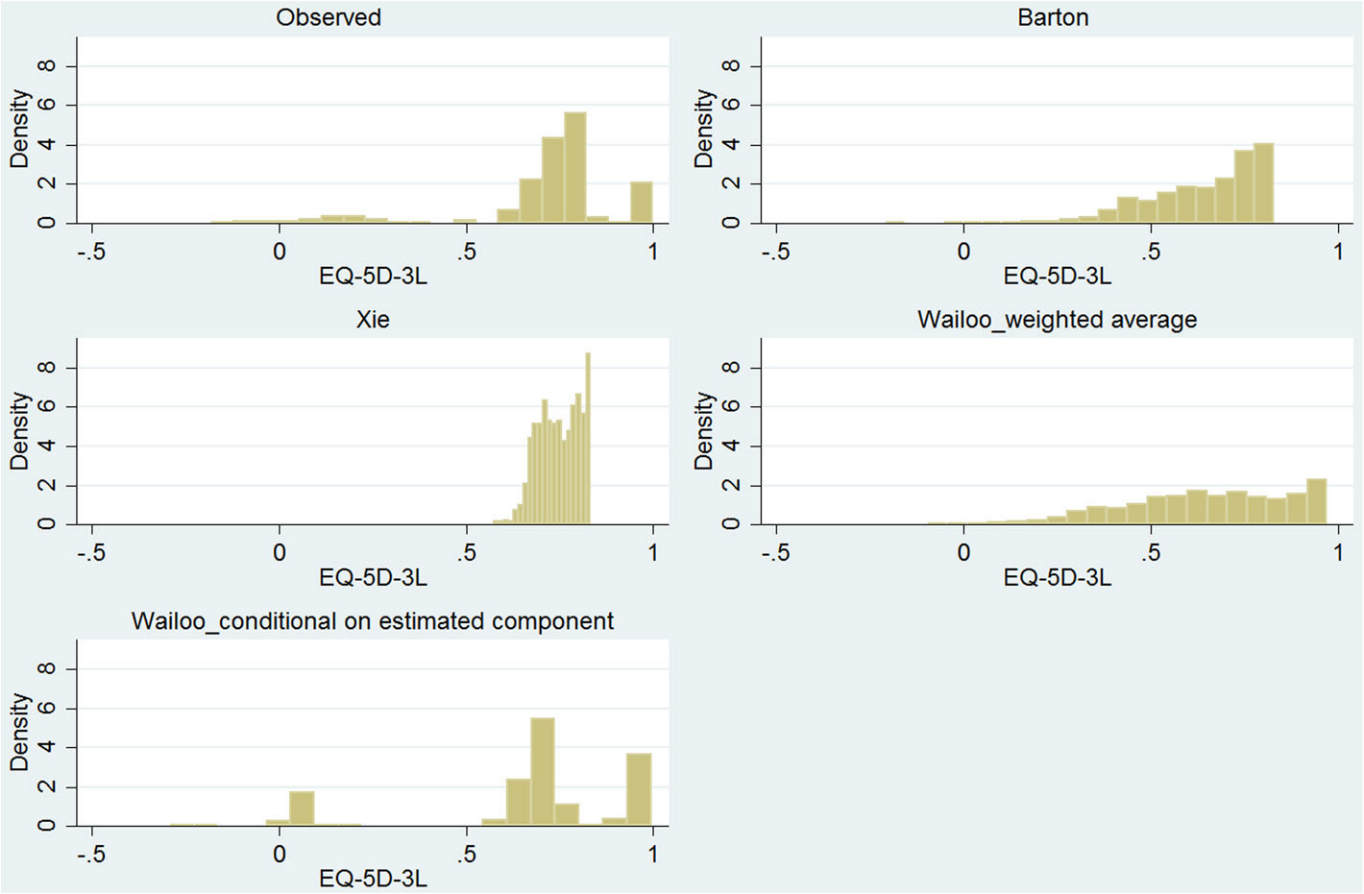
The distribution of the observed and predicted EQ-5D-3L index scores in the study sample

The Barton OLS model and Wailoo mixture model (both WA and CEC predictions) underpredicted the overall mean observed score while the Xie OLS model overpredicted it (mean errors were lower for the Xie and Wailoo CEC than two other predictions). While the range of predicted scores were narrower than the observed scores for Barton (88 %), Xie (21.9 %), and the WA prediction from Wailoo (90 %), the CEC prediction from Wailoo had a wider range (109 %). Surprisingly, none of the OLS-based models were capable of predicting any value above 0.83 and the minimum value predicted by the Xie OLS model was 0.58 (Table 2). Two OLS-based models underestimated the observed variance but the mixture model overestimated it (the WA prediction from mixture model had very similar variance to the observed one). The distribution of observed and predicted scores showed that predictions from mixture model particularly the CEC predictions more accurately capture the observed distribution compared with the OLS models (Fig. 2). The Spearman rank correlation coefficients between the observed scores and predicted scores were moderate for both individual and EQ-5D-3L health states (Additional file 1: Table S2).

**Table 2:** Summary statistics of the observed and predicted EQ-5D-3L index scores

	Mean	SD	Median	Min	Max
Observed	0.718	0.214	0.727	−0.181	1.0
Barton prediction	0.642	0.157	0.682	−0.207	0.829
Xie prediction	0.750	0.056	0.750	0.576	0.834
Wailoo prediction_WA	0.648	0.219	0.662	−0.096	0.972
Wailoo prediction_CEC	0.676	0.280	0.703	−0.290	0.995

*SD* standard deviation, *WA* weighted average, *CEC* conditional on estimated component

**Fig. 3:**
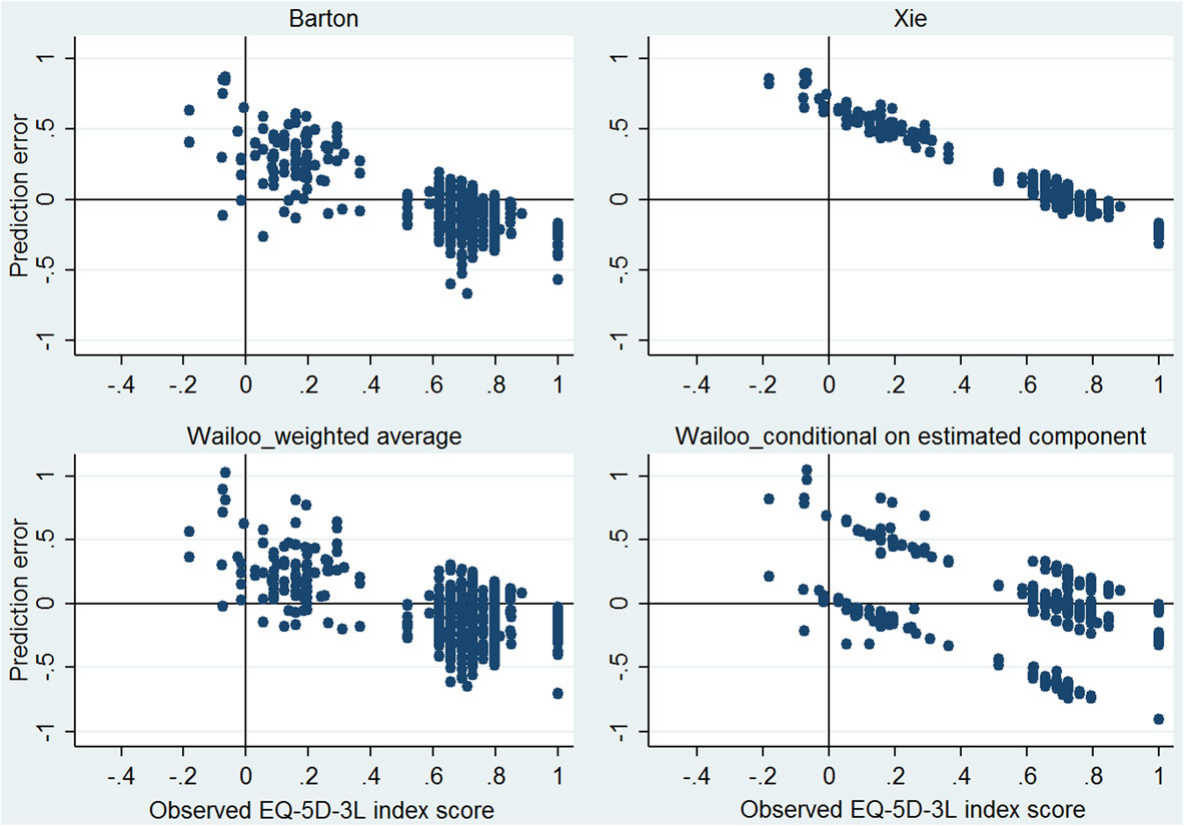
The prediction error versus the observed EQ-5D-3L index scores

While examining prediction errors across EQ-5D-3L health states revealed that all algorithms suffered from overprediction for severe health states and underprediction for mild health states, this systematic bias declined to some extend in the CEC prediction of mixture model (Fig. 3 and Additional file 2: Figure S1A). Assessing the predictive accuracy of the algorithms by levels of EQ-5D-3L index scores showed that mixture models (particularly the CEC predictions) outperformed the OLS models at the extremes of the EQ-5D-3L distribution and underperformed around the center of the distribution (Table 3). Moreover, while there were J-shape relationships between the MAE/RMSE and severity of EQ-5D-3L health state for the OLS models, the corresponding relationships were linear for mixture model (i.e., increase in MAE/RMSE as health state severity increased).

**Table 3:** Predictive accuracy of the algorithms by the observed UK EQ-5D-3L index score

EQ-5D-3L index	*n*		Barton	Xie	Wailoo	
					WA	CEC
<0.5	100	ME (95 % CI)	0.308 (0.267 to 0.349)	0.551 (0.529 to 0.574)	0.256 (0.209 to 0.303)	0.219 (0.151 to 0.288)
		MAE	0.325	0.551	0.281	0.324
		RMSE	0.372	0.563	0.350	0.410
0.5–0.699	193	ME (95 % CI)	−0.111 (−0.130 to −0.091)	0.057 (0.049 to 0.065)	−0.144 (−0.169 to −0.119)	−0.126 (−0.165 to −0.086)
		MAE	0.142	0.063	0.187	0.194
		RMSE	0.177	0.079	0.228	0.307
0.7–0.899	645	ME (95 % CI)	−0.098 (−0.106 to −0.089)	−0.013 (−0.017 to −0.009)	−0.094 (−0.106 to −0.081)	−0.054 (−0.070 to −0.038)
		MAE	0.110	0.038	0.147	0.139
		RMSE	0.147	0.048	0.187	0.211
0.9–1.0	130	ME (95 % CI)	−0.215 (−0.223 to −0.206)	−0.183 (−0.188 to −0.179)	−0.096 (−0.113 to −0.079)	−0.062 (−0.081 to −0.042)
		MAE	0.215	0.183	0.096	0.062
		RMSE	0.221	0.185	0.137	0.131
All	1068	ME (95 % CI)	−0.076 (−0.087 to −0.066)	0.032 (0.021 to 0.043)	−0.070 (−0.082 to −0.058)	−0.042 (−0.057 to −0.028)
		MAE	0.148	0.108	0.161	0.157
		RMSE	0.194	0.191	0.210	0.249
		Absolute error > |0.05 observed|, %	78.8	58.2	82.8	75.3
		Absolute error > |0.10 observed|, %	64.7	36.4	65.9	53.3
		Absolute error > |0.25 observed|, %	29.7	11.3	36.2	24.3

*CI* confidence interval, *ME* mean error, *MAE* mean absolute error, *RMSE* root mean square error, *WA* weighted average, *CEC* conditional on estimated component

**Fig. 4:**
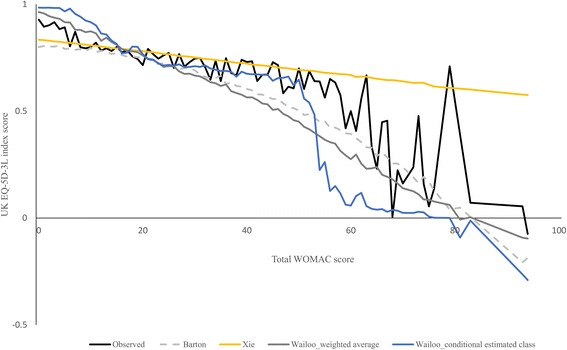
The observed and predicted mean EQ-5D-3L index scores by total WOMAC score

Assessing the predictive accuracy of the algorithms by levels of knee problems showed that the Barton OLS model underpredicted the observed EQ-5D-3L at almost all range of total WOMAC score (Fig. 4). On the other hand, the Xie OLS model overpredicted at mild knee problems (total WOMAC < 10) and overpredicted for remaining range of total WOMAC score. The mixture model overpredicted at total WOMAC scores less than 20 and underpredicted for higher levels. The MAE showed that the OLS models underperformed mixture model at mild knee problems and outperformed at most severe knee problems (Table 4). For all algorithms the lowest and highest MAE/RMSE were observed at total WOMAC score of 10–30 and 50–100, respectively. In addition, there were no statistically significant differences between the mean observed scores and WA (CEC) prediction of mixture model for total WOMAC score of 10–30 (30–50).

**Table 4:** Predictive accuracy of the algorithms by total WOMAC score interval

Total WOMAC	*n*		Barton	Xie	Wailoo	
					WA	CEC
0–10	195	ME (95 % CI)	−0.081 (−0.106 to −0.057)	−0.053 (−0.078 to −0.029)	0.048 (0.024 to 0.072)	0.093 (0.069 to 0.117)
		MAE	0.143	0.132	0.110	0.117
		RMSE	0.191	0.181	0.175	0.195
10–30	326	ME (95 % CI)	−0.021 (−0.034 to −0.009)	0.013 (0.002 to 0.025)	0.004 (−0.009 to 0.017)	0.019 (0.003 to 0.034)
		MAE	0.068	0.057	0.080	0.101
		RMSE	0.113	0.109	0.117	0.141
30–50	336	ME (95 % CI)	−0.085 (−0.104 to −0.066)	0.034 (0.016 to 0.053)	−0.119 (−0.137 to −0.100)	−0.014 (−0.033 to 0.005)
		MAE	0.160	0.093	0.184	0.112
		RMSE	0.195	0.175	0.210	0.178
50–100	211	ME (95 % CI)	−0.143 (−0.175 to −0.111)	0.135 (0.100 to 0.170)	−0.218 (−0.250 to −0.185)	−0.307 (−0.350 to −0.264)
		MAE	0.258	0.187	0.295	0.351
		RMSE	0.277	0.294	0.322	0.443

*CI* confidence interval, *ME* mean error, *MAE* mean absolute error, *RMSE* root mean square error, *WA* weighted average, *CEC* conditional on estimated component

## Discussion

The external validity of the current algorithms to estimate EQ-5D-3L from the WOMAC were, for the first time, evaluated in a truly independent sample of middle aged and elderly people with knee pain and knee OA. The result revealed that the algorithms systematically and statistically significantly overpredicted the observed scores for severe health states and underpredicted for mild health states. We also found that mixture model particularly predictions based on CEC reflect the characteristics of EQ-5D-3L distribution more accurately compared to OLS models. Moreover, while mixture model outperformed the OLS models at the extremes of the EA-5D-3L distribution, it underperformed at the middle of the distribution.

We found statistically significantly differences between the overall mean observed and predicted EQ-5D-3L index scores, even though the magnitude of prediction error particularly for the Xie OLS model and the CEC prediction of mixture model was low and can be considered acceptable in an external sample. In addition, as expected, the MAEs in our external sample was higher than values reported in the original samples (15 % larger for Barton [8] and 46 % larger for Xie [9]; no MAE was reported by Waillo [10]). However, the range of the MAEs observed in our external validation (0.108–0.161) was similar to values reported by previous mapping algorithms [2]. In line with previous studies [20, 21] we found that while the OLS models outperformed either of predictions of mixture model in the total sample, the latter performed better at the extremes of the EQ-5D-3L distribution. It should be noted that the OLS models performed better in the total sample mainly because they had better performance around the center of the observed distribution where a large proportion of our data were clustered (71.5 % of the observed EQ-5D-3L values were between 0.65 and 0.80). The tendency of predicted EQ-5D-3L values from OLS models to regress toward mean might explain this phenomenon [21] particularly for the Xie OLS model which developed using the Japanese value set [19] with a narrower range than the UK value set [18]. Interestingly, the Xie OLS model with the best performance in the total sample was not capable of predicting any EQ-5D-3L value above 0.84 and below 0.57 while 24.3 % of the observed values were distributed in these ranges. This implies that if we had more observations outside the range of 0.6 to 0.8, then the mixture model possibly outperformed the OLS models.

For all algorithms the worst predictive accuracy was observed in severe health states/knee problems implying that the predicted values for these health states should be applied with high caution regardless of the applied algorithm. More importantly, all algorithms suffered from overprediction for severe health states and underprediction for mild health states. These problems have been previously reported in the literature [2, 4, 22] and the presence of the N3 term in the UK value set and large decrement in utility due to this term has been suggested as a potential explanation [23]. Moreover, while mixture model has been applied to overcome this systematic bias [20, 21], our results showed that they can diminish it but cannot eliminate it. This systematic bias can potentially resulted in underestimating of health gain particularly for quality of life-improving interventions. While such underestimation has been reported by Barton et al. [8], the actual impact of two other mapping algorithms on health gains and cost-utility analyses should be investigated in a longitudinal study.

There are several potential explanations for observed differences between the actual and predicted EQ-5D-3L index scores. The degree of conceptual overlap between a disease-specific measure and EQ-5D-3L plays a crucial role in the strength of the algorithms [2]. As the WOMAC mainly concentrate on physical problems related to knee and do not directly capture emotional problems such as depression and anxiety, the overlap between the WOMAC and EQ-5D-3L might be limited. In mapping the Health Assessment Questionnaire (HAQ) to EQ-5D-3L, Hernández Alava et al. [20] found that adding pain as one of the main domains in the EQ-5D-3L which is not included in the HAQ summary score improved the models predictive accuracy. Therefore, adding emotional problems to the mapping algorithms of the WOMAC to EQ-5D.3L might have similar positive impact on the model fit of mapping algorithms. In addition, possible variations in overlap between the WOMAC and EQ-5D-3L across populations can influence the predictive accuracy of the mapping algorithms.

The inherent differences in populations (e.g., socio-cultural, health status, clinical practice patterns, and access to health care services) between the estimation and validation samples might partially explain the observed differences. For example, our study sample had less severe knee problems compared to the samples used in developing mapping algorithms. However, it should be highlighted that transportability of a prediction model entails that a model performs well across samples with different case mix compared to the original sample [6]. Moreover, in practice an available algorithm will be applied in variety of settings and populations and therefore assessing the transportability of mapping algorithms is important. The results of our study suggest that generalizability of the current mapping algorithms to estimate EQ-5D-3L from the WOMAC in a sample with mild knee problems is limited. Furthermore, differences induced by translation, and applied questionnaires (using the WOMAC directly versus applying the KOOS in our study) might also partially explain our findings.

The variation in performance of the mapping algorithms implies that using different algorithms might produce different QALYs and cost-utility results. Such possibility has been reported in the literature [5, 8, 24] and need to be taken into account by policy makers when making decisions based on findings from these algorithms because it is possible that pharmaceutical companies select an algorithm that support cost-effectiveness of their products. In this situations, assessing the impact of the choice of mapping algorithms on cost-effectiveness results through sensitivity analysis has been suggested [5, 24].

The current study has several limitations that should be considered when interpreting its findings. Only 48 (20 %) of 243 the possible EQ-5D-3L health states were observed in our study sample. This limits the generalizability of our findings to other patient population where the other EQ-5D-3L health states might be more common. In addition, the higher EQ-5D-3L and WOMAC scores in our sample implies that we mainly assessed the transportability of the mapping algorithms and could not assess reproducibility of them. Difference in applied method to compute the WOMAC scores (indirect calculation of the WOMAC scores using the KOOS) compared to the method applied in the mapping studies (direct application of the WOMAC) might cause differences in participants’ responses. However, the KOOS questionnaire includes the WOMAC Osteoarthritis Index LK 3.0 in its complete and original format and therefore difference might be trivial. Due to the lack of longitudinal data on changes in health status, we were not able to assess the impact of the algorithms on QALY gain and cost-effectiveness studies. In addition, this avoid possibility of assessing responsiveness and test-retest reliability of the algorithms.

## Conclusion

The current algorithms to estimate EQ-5D-3L from the WOMAC suffer from overprediction for severe health states and underprediction for mild health states which might lead to underestimation of QALY gain. While the predictions from mixture model particularly those based on classification diminish this systematic bias, they were not able to eliminate it. The mixture model outperformed the OLS models at the extremes of the EQ-5D-3L distribution and more accurately captured the characteristics of the distribution. It should be noted that our findings do not invalidate the current mapping algorithms but imply that the mapping algorithms might have limited generalizability to population with milder knee problems compared with the estimation samples.

Our results highlight the importance of external validation of the algorithms and supports the recommendations in the literature to directly collect data on utilities using preference based instruments and considering mapping algorithms as second-best solution [2, 8, 25, 26]. In addition, due to variation in performance of the mapping algorithms, we support assessing the impact of the choice of algorithms on cost-utility analysis through sensitivity analysis. Investigating the impact of the algorithms on cost-effectiveness studies, assessing their predictive accuracy in a sample with more severe knee problems, and developing a mapping algorithm for patients with mild knee problems are topics for future studies.

## Additional files

Additional file 1: Table S1. Health states and related observed and predicted EQ-5D-3L index scores. **Table S2.** Spearman’s rank correlation matrix of the observed and predicted EQ-5D-3L index scores at individual and health state level. (DOCX 18 kb)
Additional file 2: Figure S1A. The observed and predicted mean EQ-5D-3L index scores by the observed EQ-5D-3L health states. (TIF 1115 kb)
