# Supplementary material for: Assessing the external validity of algorithms to estimate EQ-5D-3L from the WOMAC
Source: Health Qual Life Outcomes. 2016 Oct 4;14:141. doi: 10.1186/s12955-016-0547-y (PMC5050671; doi:10.1186/s12955-016-0547-y)
Supplement: Additional file 1: Table S1. — Health states and related observed and predicted EQ-5D-3L index scores. Table S2. Spearman’s rank correlation matrix of the observed and predicted EQ-5D-3L index scores at individual and health state level. (DOCX 18 kb) [file 12955_2016_547_MOESM1_ESM.docx]

Table S1. Health states and related observed and predicted EQ-5D-3L index scores.

| Observed health state | UK EQ-5D index score | Barton model | | Xie model | | Wailoo model | | | |
| --- | --- | --- | --- | --- | --- | --- | --- | --- | --- |
|  |  |  |  |  |  | Weighted average | | Conditional estimated class | |
|  |  | Mean | Range | Mean | Range | Mean | Range | Mean | Range |
| 11111 (n=130) | 1.0 | 0.79 | 0.43, 0.83 | 0.82 | 0.69, 0.83 | 0.90 | 0.30, 0.97 | 0.94 | 0.1, 0.99 |
| 11112 (n=14) | 0.85 | 0.79 | 0.69, 0.83 | 0.81 | 0.75, 0.83 | 0.89 | 0.63, 0.96 | 0.92 | 0.71, 0.99 |
| 11121 (n=351) | 0.80 | 0.71 | 0.43, 0.83 | 0.77 | 0.68, 0.83 | 0.73 | 0.31, 0.96 | 0.77 | 0.06, 1.0 |
| 11122 (n=103) | 0.73 | 0.67 | 0.38, 0.83 | 0.75 | 0.67, 0.83 | 0.66 | 0.23, 0.96 | 0.70 | 0.05, 0.99 |
| 11123 (n=5) | 0.29 | 0.71 | 0.57, 0.80 | 0.78 | 0.72, 0.82 | 0.77 | 0.55, 0.93 | 0.82 | 0.69, 0.98 |
| 11131 (n=4) | 0.26 | 0.47 | 0.16, 0.62 | 0.69 | 0.63, 0.73 | 0.44 | 0.11, 0.59 | 0.51 | 0.03, 0.68 |
| 11132 (n=3) | 0.19 | 0.61 | 0.44, 0.78 | 0.75 | 0.68, 0.83 | 0.61 | 0.39, 0.97 | 0.59 | 0.08, 0.99 |
| 11211 (n=1) | 0.88 | 0.78 | - | 0.83 | - | 0.97 | - | 0.99 | - |
| 11221 (n=25) | 0.76 | 0.61 | 0.43, 0.76 | 0.73 | 0.67, 0.79 | 0.59 | 0.33, 0.86 | 0.63 | 0.05, 0.93 |
| 11222 (n=19) | 0.69 | 0.61 | 0.43, 0.82 | 0.73 | 0.67, 0.82 | 0.59 | 0.35, 0.93 | 0.61 | 0.06, 0.97 |
| 11223 (n=1) | 0.26 | 0.63 | - | 0.73 | - | 0.60 | - | 0.69 | - |
| 11233 (n=1) | −0.01 | 0.64 | - | 0.73 | - | 0.62 | - | 0.68 | - |
| 11333 (n=2) | −0.07 | 0.79 | 0.78, 0.80 | 0.80 | 0.77, 0.83 | 0.85 | 0.75, 0.96 | 0.94 | 0.90, 0.98 |
| 12121 (n=2) | 0.69 | 0.57 | 0.45, 0.70 | 0.74 | 0.69, 0.78 | 0.58 | 0.42, 0.73 | 0.71 | 0.66, 0.75 |
| 12122 (n=2) | 0.62 | 0.62 | 0.58, 0.66 | 0.73 | 0.71, 0.75 | 0.58 | 0.51, 0.65 | 0.69 | 0.67, 0.71 |
| 13121 (n=1) | 0.31 | 0.64 | - | 0.73 | - | 0.60 | - | 0.68 | - |
| 13122 (n=1) | 0.24 | 0.38 | - | 0.66 | - | 0.30 | - | 0.05 | - |
| 21111 (n=5) | 0.85 | 0.71 | 0.60, 0.82 | 0.77 | 0.72, 0.83 | 0.73 | 0.53, 0.95 | 0.76 | 0.67, 0.98 |
| 21121 (n=144) | 0.73 | 0.59 | 0.31, 0.81 | 0.73 | 0.66, 0.83 | 0.56 | 0.17, 0.97 | 0.59 | −0.01, 0.99 |
| 21122 (n=58) | 0.66 | 0.56 | 0.06, 0.80 | 0.72 | 0.61, 0.83 | 0.52 | 0.04, 0.96 | 0.53 | 0.01, 0.99 |
| 21123 (n=2) | 0.22 | 0.59 | 0.47, 0.72 | 0.72 | 0.70, 0.75 | 0.56 | 0.46, 0.65 | 0.68 | 0.68, 0.68 |
| 21131 (n=12) | 0.20 | 0.49 | 0.27, 0.68 | 0.70 | 0.64, 0.74 | 0.40 | 0.15, 0.62 | 0.42 | 0.03, 0.70 |
| 21132 (n=7) | 0.12 | 0.41 | 0.04, 0.58 | 0.68 | 0.60, 0.71 | 0.34 | −0.05, 0.57 | 0.37 | −0.19, 0.67 |
| 21211 (n=1) | 0.81 | 0.60 | - | 0.71 | - | 0.56 | - | 0.66 | - |
| 21213 (n=1) | 0.31 | 0.24 | - | 0.65 | - | 0.11 | - | 0.03 | - |
| 21221 (n=61) | 0.69 | 0.54 | 0.16, 0.82 | 0.71 | 0.63, 0.83 | 0.51 | 0.11, 0.96 | 0.57 | 0.03, 0.99 |
| 21222 (n=38) | 0.62 | 0.52 | 0.32, 0.81 | 0.70 | 0.65, 0.80 | 0.49 | 0.20, 0.88 | 0.48 | 0.03, 0.95 |
| 21223 (n=3) | 0.19 | 0.33 | 0.19, 0.45 | 0.66 | 0.63, 0.69 | 0.23 | 0.13, 0.34 | 0.05 | 0.02, 0.08 |
| 21231 (n=15) | 0.16 | 0.47 | 0.03, 0.76 | 0.70 | 0.60, 0.83 | 0.41 | −0.001, 0.97 | 0.39 | −0.02, 0.99 |
| 21232 (n=11) | 0.09 | 0.34 | 0.19, 0.54 | 0.66 | 0.63, 0.71 | 0.26 | 0.12, 0.49 | 0.15 | 0.01, 0.66 |
| 21233 (n=1) | −0.08 | 0.22 | - | 0.64 | - | 0.22 | - | 0.03 | - |
| 21313 (n=1) | 0.25 | 0.38 | - | 0.67 | - | 0.31 | - | 0.07 | - |
| 21321 (n=3) | 0.36 | 0.49 | 0.28, 0.64 | 0.69 | 0.65, 0.74 | 0.43 | 0.18, 0.57 | 0.47 | 0.03, 0.70 |
| 21331 (n=1) | 0.10 | 0.51 | - | 0.69 | - | 0.41 | - | 0.66 | - |
| 21332 (n=2) | 0.03 | 0.39 | 0.34, 0.43 | 0.67 | 0.66, 0.68 | 0.27 | 0.25, 0.29 | 0.06 | 0.04, 0.07 |
| 22121 (n=2) | 0.62 | 0.43 | 0.42, 0.44 | 0.67 | 0.67, 0.68 | 0.38 | 0.35, 0.40 | 0.34 | 0.13, 0.56 |
| 22211 (n=1) | 0.71 | 0.04 | - | 0.61 | - | 0.06 | - | 0.0 | - |
| 22221 (n=2) | 0.59 | 0.60 | 0.55, 0.64 | 0.72 | 0.71, 0.74 | 0.58 | 0.51, 0.65 | 0.68 | 0.66, 0.70 |
| 22222 (n=9) | 0.52 | 0.44 | 0.34, 0.55 | 0.68 | 0.65, 0.70 | 0.37 | 0.24, 0.51 | 0.26 | 0.03, 0.66 |
| 22223 (n=2) | 0.08 | 0.35 | 0.31, 0.38 | 0.65 | 0.63, 0.66 | 0.27 | 0.25, 0.28 | 0.02 | −0.02, 0.05 |
| 22231 (n=5) | 0.06 | 0.31 | −0.21, 0.64 | 0.67 | 0.58, 0.74 | 0.29 | −0.09, 0.63 | 0.24 | −0.26, 0.71 |
| 22232 (n=4) | −0.02 | 0.17 | −0.02, 0.28 | 0.63 | 0.60, 0.65 | 0.17 | 0.01, 0.30 | 0.02 | −0.0, 0.05 |
| 22233 (n=2) | −0.18 | 0.34 | 0.22, 0.45 | 0.66 | 0.64, 0.68 | 0.28 | 0.18, 0.39 | 0.33 | 0.03, 0.63 |
| 22321 (n=1) | 0.26 | 0.63 | - | 0.71 | - | 0.52 | - | 0.21 | - |
| 22322 (n=3) | 0.19 | 0.47 | 0.37, 0.64 | 0.69 | 0.66, 0.73 | 0.40 | 0.27, 0.63 | 0.29 | 0.04, 0.78 |
| 22332 (n=3) | −0.07 | 0.42 | −0.19, 0.78 | 0.71 | 0.58, 0.81 | 0.45 | −0.10, 0.82 | 0.39 | −0.29, 0.75 |
| 23222 (n=2) | 0.14 | 0.40 | 0.13, 0.67 | 0.67 | 0.62, 0.73 | 0.35 | 0.08, 0.61 | 0.34 | 0.0, 0.67 |
| 23223 (n=1) | −0.03 | 0.46 | - | 0.68 | - | 0.33 | - | 0.07 | - |

Table S2. Spearman’s rank correlation matrix of the observed and predicted EQ-5D-3L index scores at individual and health state level.

|  | Observed EQ-5D-3L | Barton model | Xie model | Wailoo_WA | Wailoo_CEC |
| --- | --- | --- | --- | --- | --- |
| **Individual (n=1068)** | | | | | |
| Observed EQ-5D-3L |  | 0.62 | 0.64 | 0.64 | 0.62 |
| Barton model |  |  | 0.97 | 0.96 | 0.94 |
| Xie model |  |  |  | 0.99 | 0.98 |
| Wailoo_WA |  |  |  |  | 0.97 |
| Wailoo_CDC |  |  |  |  |  |
| **Health states (n=48)** | | | | | |
| Observed EQ-5D-3L |  | 0.55 | 0.55 | 0.56 | 0.53 |
| Barton model |  |  | 0.97 | 0.98 | 0.92 |
| Xie model |  |  |  | 0.99 | 0.94 |
| Wailoo_WA |  |  |  |  | 0.95 |
| Wailoo_CDC |  |  |  |  |  |

P < 0.001 in all cases. WA: weighted average, CEC: conditional on estimated component.
