# Supplementary figures and images for: Assessing the external validity of algorithms to estimate EQ-5D-3L from the WOMAC
Source: Health Qual Life Outcomes. 2016 Oct 4;14:141. doi: 10.1186/s12955-016-0547-y (PMC5050671; doi:10.1186/s12955-016-0547-y)

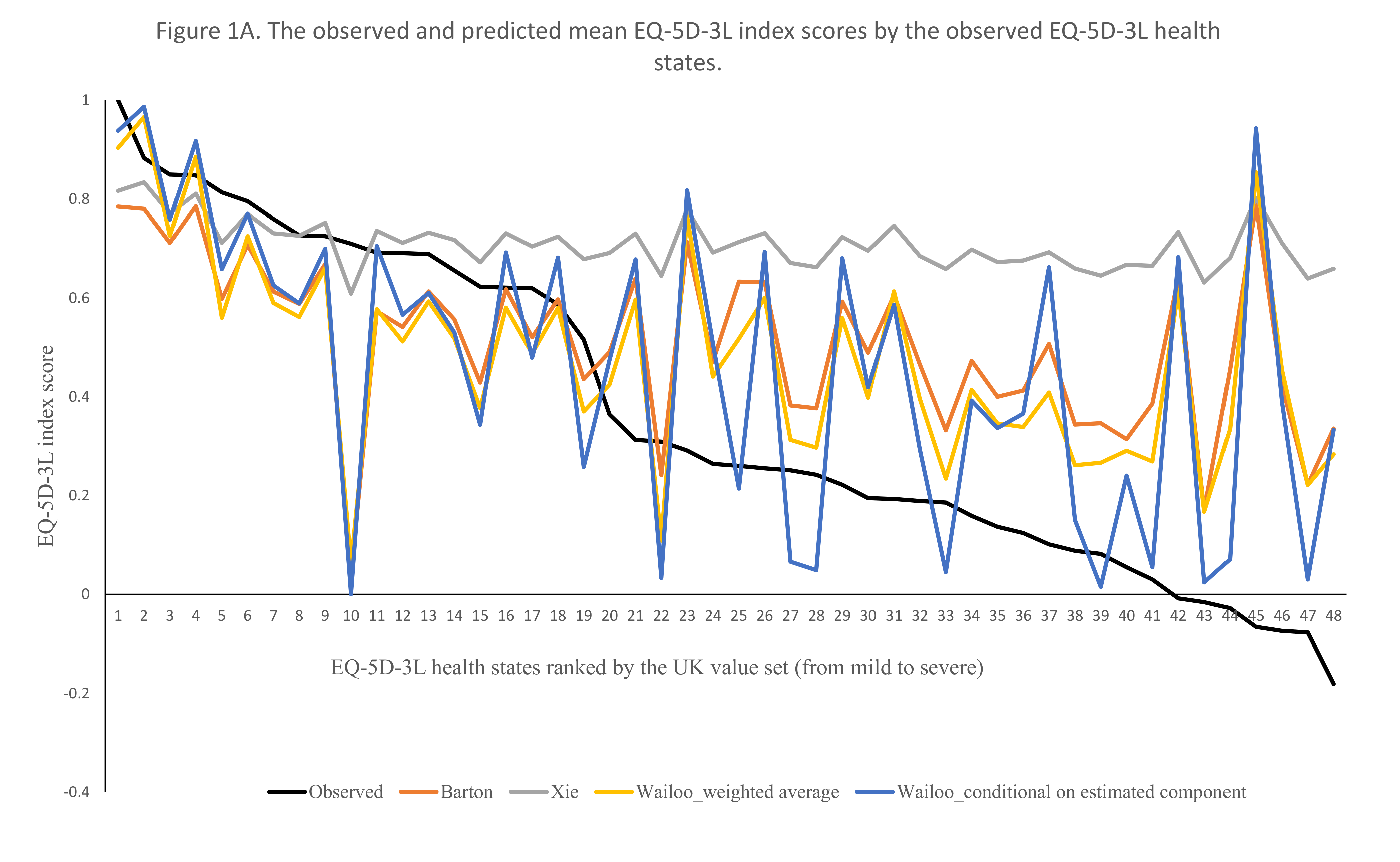

Supplement: Additional file 2: Figure S1A. — The observed and predicted mean EQ-5D-3L index scores by the observed EQ-5D-3L health states. (TIF 1115 kb) [file 12955_2016_547_MOESM2_ESM.tif]
